# Supplementary material for: The Detailed Emissions Scaling, Isolation, and Diagnostic (DESID) module in the Community Multiscale Air Quality (CMAQ) modeling system version 5.3.2
Source: Geosci Model Dev. Author manuscript; Available in PMC 2022 Jun 7. (PMC8318114; doi:10.5194/gmd-14-3407-2021)
Supplement: Supplement1 [file NIHMS1712621-supplement-Supplement1.pdf]

Supplement of Geosci. Model Dev., 14, 3383–3406, 2021
https://doi.org/10.5194/gmd-14-3383-2021-supplement
© Author(s) 2021. CC BY 4.0 License.

Geoscientific Model Development
Open Access

Supplement of

Regional CO_2 inversions with LUMIA, the Lund University Modular Inversion Algorithm, v1.0

Guillaume Monteil and Marko Scholze

Correspondence to: Guillaume Monteil (guillaume.monteil@nateko.lu.se)

The copyright of individual parts of the supplement might differ from the article licence.

Supplemental Material

Contents:

Section S1: Default Emission Control Interface for CMAQ v5.3.2 for chemical mechanism CB6r3_ae7_aq

Section S2: Emission Control Interface for implementation of Example 13 in Table 5.

Section S3: Emission Control Interface for implementation of all Examples in Table 7.

Section S4: CMAQ runscript examples for a conventional (8 offline streams) application and a high-source-resolution (46 offline streams) application.

Table S1: Examples of streams used for conventional and high-source-resolution cases.

Figure S1: a) Average 1-hr NO_2 emissions input for June 3-10, 2016. b) Change in 1-hr NO_2 emissions for CMAQ run with ECI shown in section S2. c) Average NO_2 surface concentrations predicted by CMAQ for June 3-10, 2016. d) Change in average NO_2 concentration for ECI shown in section S2.

Figure S2: a) Average 1-hr TOL emissions input for June 3-10, 2016. b) Change in 1-hr TOL emissions for CMAQ run with ECI shown in section S3. c) Average TOL surface concentrations predicted by CMAQ for June 3-10, 2016. d) Change in average TOL concentration for ECI shown in section S3.

Figure S3: a) Average 1-hr NO emissions input for June 3-10, 2016. b) Change in 1-hr NO emissions for CMAQ run with ECI shown in section S3. c) Average NO surface concentrations predicted by CMAQ for June 3-10, 2016. d) Change in average NO concentration for ECI shown in section S3.

S1: Default Emission Control Interface for CMAQ v5.3.2 for chemical mechanism CB6r3_ae7_aq

```
!------------------------------------------------------------------------------!
! EMISSION CONTROL INPUT FILE                                                  !
!  FOR THE                                                                     !
! COMMUNITY MULTISCALE AIR QUALITY (CMAQ) MODEL                                !
!  DEVELOPED AND MAINTAINED BY THE                                             !
! NATIONAL EXPOSURE RESEARCH LABORATORY, OFFICE OF RESEARCH AND DEVELOPMENT    !
! UNITED STATES ENVIRONMENTAL PROTECTION AGENCY                                !
!                                                                              !
! THIS VERSION CONSISTENT WITH THE RELEASE OF CMAQv5.3.2 (FALL 2020)           !
!------------------------------------------------------------------------------!

!------------------------------------------------------------------------------!
! Emissions Scaling Specification Section                                      !
!------------------------------------------------------------------------------!
&EmissionScalingRules
 EM_NML=
 ! Region      | Stream Label  |Emission | CMAQ-        |Phase/|Scale |Basis |Op
 !  Label      |               |Variable | Species      |Mode  |Factor|      |

                            !> DEFAULT MAPPING <!
   ! Note: Without default mapping for a species,
   !       there is no emission of that species.

   ! Default Gases
   'EVERYWHERE', 'ALL'         ,'NO2'    ,'NO2'         ,'GAS'  ,1.  ,'UNIT','a',
   'EVERYWHERE', 'ALL'         ,'NO'     ,'NO'          ,'GAS'  ,1.  ,'UNIT','a',
   'EVERYWHERE', 'ALL'         ,'HONO'   ,'HONO'        ,'GAS'  ,1.  ,'UNIT','a',
   'EVERYWHERE', 'ALL'         ,'SO2'    ,'SO2'         ,'GAS'  ,1.  ,'UNIT','a',
   'EVERYWHERE', 'ALL'         ,'NH3'    ,'NH3'         ,'GAS'  ,1.  ,'UNIT','a',
   'EVERYWHERE', 'ALL'         ,'AACD'   ,'AACD'        ,'GAS'  ,1.  ,'UNIT','a',
   'EVERYWHERE', 'ALL'         ,'ALD2'   ,'ALD2'        ,'GAS'  ,1.  ,'UNIT','a',
   'EVERYWHERE', 'ALL'         ,'FORM'   ,'FORM'        ,'GAS'  ,1.  ,'UNIT','a',
   'EVERYWHERE', 'ALL'         ,'MEOH'   ,'MEOH'        ,'GAS'  ,1.  ,'UNIT','a',
   'EVERYWHERE', 'ALL'         ,'FACD'   ,'FACD'        ,'GAS'  ,1.  ,'UNIT','a',
   'EVERYWHERE', 'ALL'         ,'CO'     ,'CO'          ,'GAS'  ,1.  ,'UNIT','a',
   'EVERYWHERE', 'ALL'         ,'ALDX'   ,'ALDX'        ,'GAS'  ,1.  ,'UNIT','a',
   'EVERYWHERE', 'ALL'         ,'ETHA'   ,'ETHA'        ,'GAS'  ,1.  ,'UNIT','a',
   'EVERYWHERE', 'ALL'         ,'ETOH'   ,'ETOH'        ,'GAS'  ,1.  ,'UNIT','a',
   'EVERYWHERE', 'ALL'         ,'KET'    ,'KET'         ,'GAS'  ,1.  ,'UNIT','a',
   'EVERYWHERE', 'ALL'         ,'PAR'    ,'PAR'         ,'GAS'  ,1.  ,'UNIT','a',
   'EVERYWHERE', 'ALL'         ,'ACET'   ,'ACET'        ,'GAS'  ,1.  ,'UNIT','a',
   'EVERYWHERE', 'ALL'         ,'PRPA'   ,'PRPA'        ,'GAS'  ,1.  ,'UNIT','a',
   'EVERYWHERE', 'ALL'         ,'ETHY'   ,'ETHY'        ,'GAS'  ,1.  ,'UNIT','a',
   'EVERYWHERE', 'ALL'         ,'ETH'    ,'ETH'         ,'GAS'  ,1.  ,'UNIT','a',
   'EVERYWHERE', 'ALL'         ,'OLE'    ,'OLE'         ,'GAS'  ,1.  ,'UNIT','a',
   'EVERYWHERE', 'ALL'         ,'IOLE'   ,'IOLE'        ,'GAS'  ,1.  ,'UNIT','a',
   'EVERYWHERE', 'ALL'         ,'ISOP'   ,'ISOP'        ,'GAS'  ,1.  ,'UNIT','a',
```

```
'EVERYWHERE', 'ALL'         ,'APIN'   ,'APIN'        ,'GAS'  ,1.  ,'UNIT','a',
'EVERYWHERE', 'ALL'         ,'TERP'   ,'TERP'        ,'GAS'  ,1.  ,'UNIT','a',
'EVERYWHERE', 'ALL'         ,'CH4'    ,'ECH4'        ,'GAS'  ,1.  ,'UNIT','a',
'EVERYWHERE', 'ALL'         ,'CL2'    ,'CL2'         ,'GAS'  ,1.  ,'UNIT','a',
'EVERYWHERE', 'ALL'         ,'HCL'    ,'HCL'         ,'GAS'  ,1.  ,'UNIT','a',
'EVERYWHERE', 'ALL'         ,'SESQ'   ,'SESQ'        ,'GAS'  ,1.  ,'UNIT','a',
'EVERYWHERE', 'ALL'         ,'SOAALK' ,'SOAALK'      ,'GAS'  ,1.  ,'UNIT','a',
'EVERYWHERE', 'ALL'        ,'ACROLEIN','ACROLEIN'    ,'GAS'  ,1.  ,'UNIT','a',
'EVERYWHERE', 'ALL'    ,'ALD2_PRIMARY','ALD2_PRIMARY','GAS'  ,1.  ,'UNIT','a',
'EVERYWHERE', 'ALL'    ,'FORM_PRIMARY','FORM_PRIMARY','GAS'  ,1.  ,'UNIT','a',
'EVERYWHERE', 'ALL'        ,'ACROLEIN','ACRO_PRIMARY','GAS'  ,1.  ,'UNIT','a',
'EVERYWHERE', 'ALL'     ,'BUTADIENE13','BUTADIENE13' ,'GAS'  ,1.  ,'UNIT','a',
'EVERYWHERE', 'ALL'         ,'SULF'   ,'SULF'        ,'GAS'  ,0.  ,'UNIT','a',
'EVERYWHERE', 'ALL'         ,'TOL'    ,'TOL'         ,'GAS'  ,1.  ,'UNIT','a',
'EVERYWHERE', 'ALL'         ,'XYLMN'  ,'XYLMN'       ,'GAS'  ,1.  ,'UNIT','a',
'EVERYWHERE', 'ALL'         ,'NAPH'   ,'NAPH'        ,'GAS'  ,1.  ,'UNIT','a',
'EVERYWHERE', 'ALL'         ,'BENZ'   ,'BENZENE'     ,'GAS'  ,1.  ,'UNIT','a',

! Default Aerosols
'EVERYWHERE', 'ALL'         ,'SULF'   ,'ASO4'        ,'FINE' ,1.  ,'MASS','a',
'EVERYWHERE', 'ALL'         ,'PSO4'   ,'ASO4'        ,'FINE' ,1.  ,'UNIT','a',
'EVERYWHERE', 'ALL'         ,'PNH4'   ,'ANH4'        ,'FINE' ,1.  ,'UNIT','a',
'EVERYWHERE', 'ALL'         ,'PNO3'   ,'ANO3'        ,'FINE' ,1.  ,'UNIT','a',
'EVERYWHERE', 'ALL'         ,'PCL'    ,'ACL'         ,'FINE' ,1.  ,'UNIT','a',
'EVERYWHERE', 'ALL'         ,'PNA'    ,'ANA'         ,'FINE' ,1.  ,'UNIT','a',
'EVERYWHERE', 'ALL'         ,'PEC'    ,'AEC'         ,'FINE' ,1.  ,'UNIT','a',
'EVERYWHERE', 'ALL'         ,'PMOTHR' ,'AOTHR'       ,'FINE' ,1.  ,'UNIT','a',
'EVERYWHERE', 'ALL'         ,'PFE'    ,'AFE'         ,'FINE' ,1.  ,'UNIT','a',
'EVERYWHERE', 'ALL'         ,'PAL'    ,'AAL'         ,'FINE' ,1.  ,'UNIT','a',
'EVERYWHERE', 'ALL'         ,'PSI'    ,'ASI'         ,'FINE' ,1.  ,'UNIT','a',
'EVERYWHERE', 'ALL'         ,'PTI'    ,'ATI'         ,'FINE' ,1.  ,'UNIT','a',
'EVERYWHERE', 'ALL'         ,'PCA'    ,'ACA'         ,'FINE' ,1.  ,'UNIT','a',
'EVERYWHERE', 'ALL'         ,'PMG'    ,'AMG'         ,'FINE' ,1.  ,'UNIT','a',
'EVERYWHERE', 'ALL'         ,'PK'     ,'AK'          ,'FINE' ,1.  ,'UNIT','a',
'EVERYWHERE', 'ALL'         ,'PMN'    ,'AMN'         ,'FINE' ,1.  ,'UNIT','a',
'EVERYWHERE', 'ALL'         ,'PH2O'   ,'AH2O'        ,'FINE' ,1.  ,'UNIT','a',

! Coarse-Mode Inorganic Ions Scaling
'EVERYWHERE', 'ALL'         ,'PMC'    ,'ACORS'   ,'COARSE',0.99675,'UNIT','a',
'EVERYWHERE', 'ALL'         ,'PMC'    ,'ASO4'    ,'COARSE',0.001  ,'UNIT','a',
'EVERYWHERE', 'ALL'         ,'PMC'    ,'ANO3'    ,'COARSE',0.00048,'UNIT','a',
'EVERYWHERE', 'ALL'         ,'PMC'    ,'ACL'     ,'COARSE',0.00145,'UNIT','a',
'EVERYWHERE', 'ALL'         ,'PMC'    ,'AH2O'    ,'COARSE',0.00032,'UNIT','a',

! Fine-Mode Primary Organic Aerosol Scaling
!   There are a series of species available for propagating emissions of primary
!   organic particles and vapor. APOC and APNCOM are nonvolatile species that
!   age chemically in the particle phase. Traditionally, all POC (primary organic
!   carbon) and PNCOM (primary non-carbon organic matter) have been represented
```

```
! by these species.
! It is more correct to use semivolatile species to account for the gas-particle
! partitioning of this POA mass. Several particle and gas-phase species are
! provided for this task, and these species vary in their volatility, which is
! quantified with the metric C*. See Donahue et al. (ES&T, 2006).
!   Gas Species :      VLVPO1  VSVPO1  VSVPO2  VSVPO3  VIVPO1
!   Particle Species : ALVPO1  ASVPO1  ASVPO2  ASVPO3  AIVPO1
!   C* (ug m-3) :       0.1      1       10     100     1000
!                 (Mostly Particle)                    (Mostly Vapor)
! To enable semivolaitle partitioning, you may direct a fraction of mass from the
! nonvolatile emission variables (POC and PNCOM) to each of these semivolatile
! CMAQ species. To conserve mass, the total of the scale factors should sum to 1.
!   Note: Each of the semivolatile species accounts for both OC and NCOM mass, so
!         rules should come in pairs (one for POC and one for PNCOM) in order to
!         conserve the total. For Example,
!          'EVERYWHERE', 'ALL' ,'POC'  ,'VSVPO2'   ,'GAS' ,0.14 ,'MASS','a',
!          'EVERYWHERE', 'ALL' ,'PNCOM','VSVPO2'   ,'GAS' ,0.14 ,'MASS','a',
!   Note: To avoid large swigns in repartitioning after emission, it's a good idea
!         to split mass between gas and particle phases, with all mass going to
!         the particle in the C* = 0.1-1 range and all gas for C* = 100-1000.
!         Species with C*=10 can generally have mass split between gas and particle
!         or be put in all gas if conditions are very clean. If too much mass
!         evaporates or condenses upon emission, the aerosol size distribution
!         will be affected.
!   Note: It is common to specify different volatility distributions for different
!         emission sources. Please use this interface to specify your model
!         parameters.
!
! --> Nonvolatile POA
 'EVERYWHERE', 'ALL'         ,'POC'    ,'APOC'       ,'FINE',0.   ,'MASS','a',
 'EVERYWHERE', 'ALL'         ,'PNCOM'  ,'APNCOM'     ,'FINE',0.   ,'MASS','a',
! --> Semivolatile POA
 'EVERYWHERE', 'ALL'         ,'POC'    ,'VLVPO1'     ,'GAS' ,0.   ,'MASS','a',
 'EVERYWHERE', 'ALL'         ,'PNCOM'  ,'VLVPO1'     ,'GAS' ,0.   ,'MASS','a',
 'EVERYWHERE', 'ALL'         ,'POC'    ,'VSVPO1'     ,'GAS' ,0.045,'MASS','a',
 'EVERYWHERE', 'ALL'         ,'PNCOM'  ,'VSVPO1'     ,'GAS' ,0.045,'MASS','a',
 'EVERYWHERE', 'ALL'         ,'POC'    ,'VSVPO2'     ,'GAS' ,0.14 ,'MASS','a',
 'EVERYWHERE', 'ALL'         ,'PNCOM'  ,'VSVPO2'     ,'GAS' ,0.14 ,'MASS','a',
 'EVERYWHERE', 'ALL'         ,'POC'    ,'VSVPO3'     ,'GAS' ,0.18 ,'MASS','a',
 'EVERYWHERE', 'ALL'         ,'PNCOM'  ,'VSVPO3'     ,'GAS' ,0.18 ,'MASS','a',
 'EVERYWHERE', 'ALL'         ,'POC'    ,'VIVPO1'     ,'GAS' ,0.50 ,'MASS','a',
 'EVERYWHERE', 'ALL'         ,'PNCOM'  ,'VIVPO1'     ,'GAS' ,0.50 ,'MASS','a',
 'EVERYWHERE', 'ALL'         ,'POC'    ,'ALVPO1'     ,'FINE',0.09 ,'MASS','a',
 'EVERYWHERE', 'ALL'         ,'PNCOM'  ,'ALVPO1'     ,'FINE',0.09 ,'MASS','a',
 'EVERYWHERE', 'ALL'         ,'POC'    ,'ASVPO1'     ,'FINE',0.045,'MASS','a',
 'EVERYWHERE', 'ALL'         ,'PNCOM'  ,'ASVPO1'     ,'FINE',0.045,'MASS','a',
 'EVERYWHERE', 'ALL'         ,'POC'    ,'ASVPO2'     ,'FINE',0.   ,'MASS','a',
 'EVERYWHERE', 'ALL'         ,'PNCOM'  ,'ASVPO2'     ,'FINE',0.   ,'MASS','a',
 'EVERYWHERE', 'ALL'         ,'POC'    ,'ASVPO3'     ,'FINE',0.   ,'MASS','a',
```

```
 'EVERYWHERE', 'ALL'         ,'PNCOM'  ,'ASVPO3'     ,'FINE',0.   ,'MASS','a',
 'EVERYWHERE', 'ALL'         ,'POC'    ,'AIVPO1'     ,'FINE',0.   ,'MASS','a',
 'EVERYWHERE', 'ALL'         ,'PNCOM'  ,'AIVPO1'     ,'FINE',0.   ,'MASS','a',

! pcSOA is a CMAQ species introduced to account for missing pathways for SOA
! formation from combustion sources. It includes IVOC oxidation as well as other
! phenomena (Murphy et al., ACP, 2017). It was parameterized primarily in LA,
! where vehicle exhaust continues to dominate.
 'EVERYWHERE', 'ALL'          ,'POC'   ,'PCVOC'      ,'GAS' ,6.579,'MASS','a',
 'EVERYWHERE', 'ALL'          ,'PNCOM' ,'PCVOC'      ,'GAS' ,6.579,'MASS','a',
! However, the added pcSOA is probably inappropriate for Fire sources, especially
! in its current configuration. This pathway should be zeroed out for all fire
! and wood-burning related sources.
 'EVERYWHERE', 'PT_FIRES'     ,'ALL'   ,'PCVOC'      ,'GAS' ,0.0  ,'MASS','o',
 'EVERYWHERE', 'PT_RXFIRES'   ,'ALL'   ,'PCVOC'      ,'GAS' ,0.0  ,'MASS','o',
 'EVERYWHERE', 'PT_AGFIRES'   ,'ALL'   ,'PCVOC'      ,'GAS' ,0.0  ,'MASS','o',
 'EVERYWHERE', 'PT_OTHFIRES'  ,'ALL'   ,'PCVOC'      ,'GAS' ,0.0  ,'MASS','o',
 'EVERYWHERE', 'PT_FIRES_MXCA','ALL'   ,'PCVOC'      ,'GAS' ,0.0  ,'MASS','o',
 'EVERYWHERE', 'GR_RES_FIRES' ,'ALL'   ,'PCVOC'      ,'GAS' ,0.0  ,'MASS','o',

! Wind-Blown Dust and Sea Spray Scaling
! Fine Components
'EVERYWHERE', 'ALL'    ,'PMFINE_SO4'  ,'ASO4'        ,'FINE',1.   ,'UNIT','a',
'EVERYWHERE', 'ALL'    ,'PMFINE_NO3'  ,'ANO3'        ,'FINE',1.   ,'UNIT','a',
'EVERYWHERE', 'ALL'    ,'PMFINE_CL'   ,'ACL'         ,'FINE',1.   ,'UNIT','a',
'EVERYWHERE', 'ALL'    ,'PMFINE_NH4'  ,'ANH4'        ,'FINE',1.   ,'UNIT','a',
'EVERYWHERE', 'ALL'    ,'PMFINE_NA'   ,'ANA'         ,'FINE',1.   ,'UNIT','a',
'EVERYWHERE', 'ALL'    ,'PMFINE_CA'   ,'ACA'         ,'FINE',1.   ,'UNIT','a',
'EVERYWHERE', 'ALL'    ,'PMFINE_MG'   ,'AMG'         ,'FINE',1.   ,'UNIT','a',
'EVERYWHERE', 'ALL'    ,'PMFINE_K'    ,'AK'          ,'FINE',1.   ,'UNIT','a',
'EVERYWHERE', 'ALL'    ,'PMFINE_FE'   ,'AFE'         ,'FINE',1.   ,'UNIT','a',
'EVERYWHERE', 'ALL'    ,'PMFINE_AL'   ,'AAL'         ,'FINE',1.   ,'UNIT','a',
'EVERYWHERE', 'ALL'    ,'PMFINE_SI'   ,'ASI'         ,'FINE',1.   ,'UNIT','a',
'EVERYWHERE', 'ALL'    ,'PMFINE_TI'   ,'ATI'         ,'FINE',1.   ,'UNIT','a',
'EVERYWHERE', 'ALL'    ,'PMFINE_MN'   ,'AMN'         ,'FINE',1.   ,'UNIT','a',
'EVERYWHERE', 'ALL'    ,'PMFINE_H2O'  ,'AH2O'        ,'FINE',1.   ,'UNIT','a',
'EVERYWHERE', 'ALL'    ,'PMFINE_OTHR' ,'AOTHR'       ,'FINE',1.   ,'UNIT','a',

! :Scaling of Fine-Mode POA from Wind-Blown Dust or Sea Spray. Either the
! :Nonvolatile POA should be propagated to the transport model, or the Low
! :Volatility POA should be propagated, not both.
! :  --> Nonvolatile POA
   !'EVERYWHERE', 'ALL'    ,'PMFINE_POC' ,'APOC'         ,'FINE',1.   ,'UNIT','a',
   !'EVERYWHERE', 'ALL'   ,'PMFINE_PNCOM','APNCOM'       ,'FINE',1.   ,'UNIT','a',
! :  --> Semivolatile POA
   'EVERYWHERE', 'ALL'    ,'PMFINE_LVPO1','ALVPO1'      ,'FINE',1.   ,'UNIT','a',
   'EVERYWHERE', 'ALL'    ,'PMFINE_LVOO1','ALVOO1'      ,'FINE',1.   ,'UNIT','a',

! Wind-Blown Dust and Sea Spray Scaling
```

```
   ! Coarse Components
   'EVERYWHERE', 'ALL'    ,'PMCOARSE_SO4','ASO4'      ,'COARSE',1.   ,'UNIT','a',
   'EVERYWHERE', 'ALL'    ,'PMCOARSE_NO3','ANO3'      ,'COARSE',1.   ,'UNIT','a',
   'EVERYWHERE', 'ALL'    ,'PMCOARSE_CL' ,'ACL'       ,'COARSE',1.   ,'UNIT','a',
   'EVERYWHERE', 'ALL'    ,'PMCOARSE_H2O','AH2O'      ,'COARSE',1.   ,'UNIT','a',
   'EVERYWHERE', 'ALL'   ,'PMCOARSE_SOIL','ASOIL'     ,'COARSE',1.   ,'UNIT','a',
   'EVERYWHERE', 'ALL' ,'PMCOARSE_SEACAT','ASEACAT'   ,'COARSE',1.   ,'UNIT','a',
/

!------------------------------------------------------------------------------!
! Size Distribution Specification Section                                      !
!   Each size distribution rule either modifies the parameters associated with !
!   the aerosol modes of a particular stream, or adds new modes to a particular!
!   stream if they do not already exist.                                       !
!------------------------------------------------------------------------------!

&SizeDistributions
 SD_NML    =
 !         | Stream Label   | Surr. Mode   | Ref. Mode
 !<Default>  'ALL'          ,'FINE'        ,'FINE_REF',
 !<Default>  'ALL'          ,'COARSE'      ,'COARSE_REF',
             'WBDUST'       ,'FINE'        ,'FINE_WBDUST',
             'WBDUST'       ,'COARSE'      ,'COARSE_WBDUST',
             'SEASPRAY'     ,'FINE'        ,'FINE_SEASPRAY',
             'SEASPRAY'     ,'COARSE'      ,'COARSE_SEASPRAY',
 !<Example>  'AIRCRAFT'     ,'FINE'        ,'AIR_FINE',   !To use these examples, you
 !<Example>  'AIRCRAFT'     ,'COARSE'      ,'AIR_COARSE', ! must add entries for AIR_FINE
                                                          !  and  AIR_COARSE  to  the  data
structure
                                                          ! em_aero_ref in AERO_DATA.

/

!------------------------------------------------------------------------------!
! Region-Based Scaling Specification Section                                   !
!   It is possible in CMAQ to scale emissions for a subset of the model domain !
!   using gridded masks to indicate where the scaling should occur. These masks!
!   should be of type real and provided as variables on a file with format     !
!   consistent with IO-API. Any number of files and variables may be used to   !
!   specify 1 or more "regions" to be used in CMAQ. This section of the name-  !
!   list provides users with an interface to name these regions and identify   !
!   the stream data for each.
!------------------------------------------------------------------------------!

&RegionsRegistry
 RGN_NML  =
 !          | Region Label   | File_Label  | Variable on File
 !<Default>    'EVERYWHERE'  ,'N/A'        ,'N/A',
 !<Example>    'WATER'       ,'CMAQ_MASKS' ,'OPEN',
```

```
 !<Example>    'ALL'         ,'CMAQ_MASKS' ,'ALL',
 !<Example>    'ALL'         ,'ISAM_REGIONS','ALL',
/

!------------------------------------------------------------------------------!
! Emissions Scaling Family Definitions                                         !
!    This section includes definitions for families of CMAQ chemical species,  !
!    emission streams and region combinations. Please see the Emissions        !
!    Scaling Specification Section for a definitions of CMAQ species, Regions, !
!    and Streams. For each type of family, please indicate the number of       !
!    families you are prescribing (e.g. NChemFamilies=1). Then for each Family !
!    indicate the Name, the number of components, and the name of each         !
!    component. All entries are case-insensitive. See the Emissions tutorial   !
!    in the CMAQ Repository for detailed directions for how to work with       !
!    Families.                                                                 !
!------------------------------------------------------------------------------!

!&ChemicalFamilies
! NChemFamilies         = 1
! ChemFamilyName(1)     = 'NOX'
! ChemFamilyNum(1)      = 2
! ChemFamilyMembers(1,:)= 'NO','NO2'
!/

!&StreamFamilies
! NStreamFamilies         = 1
! StreamFamilyName(1)     = 'PT_SOURCES'
! StreamFamilyNum(1)      = 3
! StreamFamilyMembers(1,:)= 'PT_NONEGU','PT_EGU','PT_OTHER'
!/

!&RegionFamilies
! NRegionFamilies         = 1
! RegionFamilyName(1)     = 'Water'
! RegionFamilyNum(1)      = 2
! RegionFamilyMembers(1,:)= 'SURF','OPEN'
!/
```

S2: Emission Control Interface for implementation of Example 13 in Table 5.

Two lines (one comment and one rule) are added to the default ECI. Three lines above and below are repeated from the default ECI in this example to illustrate the location of the added lines.

```
…
   'EVERYWHERE', 'ALL'         ,'XYLMN'  ,'XYLMN'       ,'GAS'  ,1.  ,'UNIT','a',
   'EVERYWHERE', 'ALL'         ,'NAPH'   ,'NAPH'        ,'GAS'  ,1.  ,'UNIT','a',
   'EVERYWHERE', 'ALL'         ,'BENZ'   ,'BENZENE'     ,'GAS'  ,1.  ,'UNIT','a',

   ! Scaling Modification from Table 5 (Example 13)
   'IL',         'ALL'             ,'NO2','NO2'         ,'GAS'  ,1.1 ,'UNIT','o',

   ! Default Aerosols
   'EVERYWHERE', 'ALL'         ,'SULF'   ,'ASO4'        ,'FINE' ,1.  ,'MASS','a',
   'EVERYWHERE', 'ALL'         ,'PSO4'   ,'ASO4'        ,'FINE' ,1.  ,'UNIT','a',
…
```

S3: Emission Control Interface for implementation of all Examples in Table 7.

Seven rules and two comments are added to the rule section of the default ECI. Three lines are repeated below from the default ECI rule section to illustrate the location of the added rules. Region and family specifications appear after in their corresponding sections.

```
…
   'EVERYWHERE', 'ALL'        ,'ACROLEIN','ACRO_PRIMARY','GAS'  ,1.  ,'UNIT','a',
   'EVERYWHERE', 'ALL'     ,'BUTADIENE13','BUTADIENE13' ,'GAS'  ,1.  ,'UNIT','a',
   'EVERYWHERE', 'ALL'         ,'SULF'   ,'SULF'        ,'GAS'  ,0.  ,'UNIT','a',

   ! Mapping Rules from Table 7 (Examples 15-18)
   'EVERYWHERE', 'ALL'         ,'TOL'    ,'TOL'         ,'GAS'  ,1.  ,'UNIT','a',
   'EVERYWHERE', 'ALL'         ,'XYLMN'  ,'XYLMN'       ,'GAS'  ,1.  ,'UNIT','a',
   'EVERYWHERE', 'ALL'         ,'NAPH'   ,'NAPH'        ,'GAS'  ,1.  ,'UNIT','a',
   'EVERYWHERE', 'ALL'         ,'BENZ'   ,'BENZENE'     ,'GAS'  ,1.  ,'UNIT','a',

   ! Scaling Modifications from Table 7 (Examples 19-21)
   'EVERYWHERE', 'ALL'         ,'ALL'    ,'AROMATICS'   ,'GAS'  ,0.6  ,'UNIT','m',
   'SOUTHWEST',  'ALL'         ,'NO'     ,'NO'          ,'GAS'  ,0.9  ,'UNIT','o',
   'EVERYWHERE', 'INDUS'       ,'ALL'    ,'AROMATICS'   ,'GAS'  ,0.3  ,'UNIT','o',

   ! Default Aerosols
   'EVERYWHERE', 'ALL'         ,'SULF'   ,'ASO4'        ,'FINE' ,1.  ,'MASS','a',
   'EVERYWHERE', 'ALL'         ,'PSO4'   ,'ASO4'        ,'FINE' ,1.  ,'UNIT','a',
…

!------------------------------------------------------------------------------!
! Region-Based Scaling Specification Section                                   !
!   It is possible in CMAQ to scale emissions for a subset of the model domain !
!   using gridded masks to indicate where the scaling should occur. These masks!
!   should be of type real and provided as variables on a file with format     !
!   consistent with IO-API. Any number of files and variables may be used to  !
!   specify 1 or more "regions" to be used in CMAQ. This section of the name-  !
!   list provides users with an interface to name these regions and identify   !
!   the stream data for each.
!------------------------------------------------------------------------------!

&RegionsRegistry
 RGN_NML  =
 !          | Region Label   | File_Label  | Variable on File

               'ALL'         ,'US_STATES'  ,'ALL',
/

!------------------------------------------------------------------------------!
! Emissions Scaling Family Definitions                                         !
!    This section includes definitions for families of CMAQ chemical species,  !
!    emission streams and region combinations. Please see the Emissions        !
!    Scaling Specification Section for a definitions of CMAQ species, Regions, !
!    and Streams. For each type of family, please indicate the number of       !
```

```
!    families you are prescribing (e.g. NChemFamilies=1). Then for each Family !
!    indicate the Name, the number of components, and the name of each        !
!    component. All entries are case-insensitive. See the Emissions tutorial   !
!    in the CMAQ Repository for detailed directions for how to work with       !
!    Families.                                                                 !
!------------------------------------------------------------------------------!

&ChemicalFamilies
 NChemFamilies         = 1
 ChemFamilyName(1)     = 'AROMATICS'
 ChemFamilyNum(1)      = 4
 ChemFamilyMembers(1,:)= 'TOL','XYLMN','BENZENE','NAPH'
/

&StreamFamilies
 NStreamFamilies         = 1
 StreamFamilyName(1)     = 'INDUS'
 StreamFamilyNum(1)      = 3
 StreamFamilyMembers(1,:)= 'POINT_NONEGU','POINT_EGU','POINT_OTHER'
/

&RegionFamilies
 NRegionFamilies         = 1
 RegionFamilyName(1)     = 'SOUTHWEST'
 RegionFamilyNum(1)      = 5
 RegionFamilyMembers(1,:)= 'CA','NM','AZ','NV','UT'
/
```

S4. CMAQ runscript examples for a conventional (10 offline streams) application and a high-source-resolution (46 offline streams) application.

<u>Relevant portion of conventional CMAQ runscript specification with 2 offline gridded and 8 offline point streams</u>

```
#> Emissions Control File
setenv EMISSCTRL_NML EmissCtrl_cb6r3_ae7_aq.nml

#> Spatial Masks For Emissions Scaling
setenv CMAQ_MASKS 12US1_states.nc #> horizontal grid-dependent geographic masks for states

#> Set default for symbolic date override
setenv EMIS_SYM_DATE F

#> Gridded Emissions Files
setenv N_EMIS_GR 2

# Emission Rates for Gridded Sources
setenv GR_EMIS_001 ${EMISpath}/emis_mole_all_20160101_cb6_bench.nc
setenv GR_EMIS_002 ${EMISpath2}/emis_mole_rwc_20160101_12US1_cmaq_cb6_2016ff_16j.nc

# Label Each Gridded Emission Stream
setenv GR_EMIS_LAB_001 GRIDDED_EMIS
setenv GR_EMIS_LAB_002 GR_RES_FIRES

# Enforce error if Gridded Emission File Date does not Match Model Date
setenv GR_EM_SYM_DATE_001 F # To change default behaviour please see Users Guide for EMIS_SYM_DATE
setenv GR_EM_SYM_DATE_002 F # To change default behaviour please see Users Guide for EMIS_SYM_DATE

#> Offline point emissions configuration
setenv N_EMIS_PT 8          #> Number of elevated source groups

# Time-Independent Stack Parameters for Offline Point Sources
setenv STK_GRPS_001 $IN_PTpath/stack_groups/stack_groups_ptnonipm.nc
setenv STK_GRPS_002 $IN_PTpath/stack_groups/stack_groups_ptegu.nc
setenv STK_GRPS_003 $IN_PTpath/stack_groups/stack_groups_othpt.nc
setenv STK_GRPS_004 $IN_PTpath/stack_groups/stack_groups_ptagfire_20160101.nc
setenv STK_GRPS_005 $IN_PTpath/stack_groups/stack_groups_ptfire_20160101.nc
setenv STK_GRPS_006 $IN_PTpath/stack_groups/stack_groups_ptfire_othna_20160101.nc
setenv STK_GRPS_007 $IN_PTpath/stack_groups/stack_groups_pt_oilgas.nc
setenv STK_GRPS_008 $IN_PTpath/stack_groups/stack_groups_cmv_c3.nc

# Emission Rates for Offline Point Sources
setenv STK_EMIS_001 $IN_PTpath/ptnonipm/inln_mole_ptnonipm_20160101.nc
setenv STK_EMIS_002 $IN_PTpath/ptegu/inln_mole_ptegu_20160101.nc
setenv STK_EMIS_003 $IN_PTpath/othpt/inln_mole_othpt_20160101.nc
setenv STK_EMIS_004 $IN_PTpath/ptagfire/inln_mole_ptagfire_20160101.nc
setenv STK_EMIS_005 $IN_PTpath/ptfire/inln_mole_ptfire_20160101.nc
setenv STK_EMIS_006 $IN_PTpath/ptfire_othna/inln_mole_ptfire_othna_20160101.nc
```

```
setenv STK_EMIS_007 $IN_PTpath/pt_oilgas/inln_mole_pt_oilgas_20160101.nc
setenv STK_EMIS_008 $IN_PTpath/cmv_c3/inln_mole_cmv_c3_20160101.nc

 # Label Each Offline Point Emissions Stream
setenv STK_EMIS_LAB_001 PT_NONEGU
setenv STK_EMIS_LAB_002 PT_EGU
setenv STK_EMIS_LAB_003 PT_OTHER
setenv STK_EMIS_LAB_004 PT_AGFIRES
setenv STK_EMIS_LAB_005 PT_FIRES
setenv STK_EMIS_LAB_006 PT_OTHFIRES
setenv STK_EMIS_LAB_007 PT_OILGAS
setenv STK_EMIS_LAB_008 PT_CMV

# Allow CMAQ to Use Point Source files with dates that do not match the internal model date
# To change default behaviour please see Users Guide for EMIS_SYM_DATE
setenv STK_EM_SYM_DATE_001 T
setenv STK_EM_SYM_DATE_002 T
setenv STK_EM_SYM_DATE_003 T
setenv STK_EM_SYM_DATE_004 T
setenv STK_EM_SYM_DATE_005 T
setenv STK_EM_SYM_DATE_006 T
setenv STK_EM_SYM_DATE_007 T
setenv STK_EM_SYM_DATE_008 T
```

Relevant portion of High-source-resolution CMAQ runscript specification with 27 offline gridded and 19 offline point streams

```
#> Emissions Control File
setenv EMISSCTRL_NML EmissCtrl_cb6r3_ae7_aq.nml

#> Spatial Masks For Emissions Scaling
setenv CMAQ_MASKS 12US1_states.nc #> horizontal grid-dependent geographic masks for states

#> Set default for symbolic date override
#> No need for stream-specific symbolic date overrides below
setenv EMIS_SYM_DATE T

#> Gridded Emissions Files
setenv N_EMIS_GR 27

# Emission Rates for Gridded Sources
setenv GR_EMIS_001 ${GR_EMISpath}/agfire/emis_mole_agfire_20160101_12US1.ncf
setenv GR_EMIS_002 ${GR_EMISpath}/agriculture/emis_mole_agriculture_20160101_12US1.ncf
setenv GR_EMIS_003 ${GR_EMISpath}/aircraft/emis_mole_aircraft_20160101_12US1.ncf
setenv GR_EMIS_004 ${GR_EMISpath}/cooking/emis_mole_cooking_20160101_12US1.ncf
setenv GR_EMIS_005 ${GR_EMISpath}/dust/emis_mole_dust_20160101_12US1.ncf
setenv GR_EMIS_006 ${GR_EMISpath}/egu_biomass/emis_mole_egu_biomass_20160101_12US1.ncf
setenv GR_EMIS_007 ${GR_EMISpath}/egu_coal/emis_mole_egu_coal_20160101_12US1.ncf
setenv GR_EMIS_008 ${GR_EMISpath}/egu_natgas/emis_mole_egu_natgas_20160101_12US1.ncf
setenv GR_EMIS_009 ${GR_EMISpath}/egu_oil/emis_mole_egu_oil_20160101_12US1.ncf
setenv GR_EMIS_010 ${GR_EMISpath}/indus_biomass/emis_mole_indus_biomass_20160101_12US1.ncf
setenv GR_EMIS_011 ${GR_EMISpath}/indus_coal/emis_mole_indus_coal_20160101_12US1.ncf
setenv GR_EMIS_012 ${GR_EMISpath}/indus_gasoline/emis_mole_indus_gasoline_20160101_12US1.ncf
setenv GR_EMIS_013 ${GR_EMISpath}/indus_natgas/emis_mole_indus_natgas_20160101_12US1.ncf
setenv GR_EMIS_014 ${GR_EMISpath}/indus_oil/emis_mole_indus_oil_20160101_12US1.ncf
setenv GR_EMIS_015 ${GR_EMISpath}/indus_wood/emis_mole_indus_wood_20160101_12US1.ncf
setenv GR_EMIS_016 ${GR_EMISpath}/marine/emis_mole_marine_20160101_12US1.ncf
setenv GR_EMIS_017 ${GR_EMISpath}/nonroad_diesel/emis_mole_nonroad_diesel_20160101_12US1.ncf
setenv GR_EMIS_018 ${GR_EMISpath}/nonroad_gas/emis_mole_nonroad_gas_20160101_12US1.ncf
setenv GR_EMIS_019 ${GR_EMISpath}/onroad_diesel/emis_mole_onroad_diesel_20160101_12US1.ncf
setenv GR_EMIS_020 ${GR_EMISpath}/onroad_gas/emis_mole_onroad_gas_20160101_12US1.ncf
setenv GR_EMIS_021 ${GR_EMISpath}/other/emis_mole_other_20160101_12US1.ncf
setenv GR_EMIS_022 ${GR_EMISpath}/rail/emis_mole_rail_20160101_12US1.ncf
setenv GR_EMIS_023 ${GR_EMISpath}/res_diesel/emis_mole_res_diesel_20160101_12US1.ncf
setenv GR_EMIS_024 ${GR_EMISpath}/res_openfire/emis_mole_res_openfire_20160101_12US1.ncf
setenv GR_EMIS_025 ${GR_EMISpath}/res_wood/emis_mole_res_wood_20160101_12US1.ncf
setenv GR_EMIS_026 ${GR_EMISpath}/rxfire/emis_mole_rxfire_20160101_12US1.ncf
setenv GR_EMIS_027 ${GR_EMISpath}/wildfire/emis_mole_wildfire_20160101_12US1.ncf

# Label Each Gridded Emission Stream
setenv GR_EMIS_LAB_001 GR_AGFIRE
setenv GR_EMIS_LAB_002 GR_AGRICULTURE
setenv GR_EMIS_LAB_003 GR_AIRCRAFT
setenv GR_EMIS_LAB_004 GR_COOKING
```

```
setenv GR_EMIS_LAB_005 GR_DUST
setenv GR_EMIS_LAB_006 GR_EGU_BIOMASS
setenv GR_EMIS_LAB_007 GR_EGU_COAL
setenv GR_EMIS_LAB_008 GR_EGU_NATGAS
setenv GR_EMIS_LAB_009 GR_EGU_OIL
setenv GR_EMIS_LAB_010 GR_INDUS_BIOMASS
setenv GR_EMIS_LAB_011 GR_INDUS_COAL
setenv GR_EMIS_LAB_012 GR_INDUS_GASOLINE
setenv GR_EMIS_LAB_013 GR_INDUS_NATGAS
setenv GR_EMIS_LAB_014 GR_INDUS_OIL
setenv GR_EMIS_LAB_015 GR_INDUS_WOOD
setenv GR_EMIS_LAB_016 GR_MARINE
setenv GR_EMIS_LAB_017 GR_NONROAD_DIESEL
setenv GR_EMIS_LAB_018 GR_NONROAD_GAS
setenv GR_EMIS_LAB_019 GR_ONROAD_DIESEL
setenv GR_EMIS_LAB_020 GR_ONROAD_GAS
setenv GR_EMIS_LAB_021 GR_OTHER
setenv GR_EMIS_LAB_022 GR_RAIL
setenv GR_EMIS_LAB_023 GR_RES_DIESEL
setenv GR_EMIS_LAB_024 GR_RES_OPENFIRE
setenv GR_EMIS_LAB_025 GR_RES_WOOD
setenv GR_EMIS_LAB_026 GR_RXFIRE
setenv GR_EMIS_LAB_027 GR_WILDFIRE

#> Offline Point Emissions Files
setenv N_EMIS_PT 19          #> Number of elevated source groups

# Time-Independent Stack Parameters for Inline Point Sources
setenv STK_GRPS_001 $ST_EMISpath/aircraft/stack_groups_aircraft.ncf
setenv STK_GRPS_002 $ST_EMISpath/dust/stack_groups_dust.ncf
setenv STK_GRPS_003 $ST_EMISpath/egu_biomass/stack_groups_egu_biomass.ncf
setenv STK_GRPS_004 $ST_EMISpath/egu_coal/stack_groups_egu_coal.ncf
setenv STK_GRPS_005 $ST_EMISpath/egu_natgas/stack_groups_egu_natgas.ncf
setenv STK_GRPS_006 $ST_EMISpath/egu_oil/stack_groups_egu_oil.ncf
setenv STK_GRPS_007 $ST_EMISpath/indus_biomass/stack_groups_indus_biomass.ncf
setenv STK_GRPS_008 $ST_EMISpath/indus_coal/stack_groups_indus_coal.ncf
setenv STK_GRPS_009 $ST_EMISpath/indus_gasoline/stack_groups_indus_gasoline.ncf
setenv STK_GRPS_010 $ST_EMISpath/indus_natgas/stack_groups_indus_natgas.ncf
setenv STK_GRPS_011 $ST_EMISpath/indus_oil/stack_groups_indus_oil.ncf
setenv STK_GRPS_012 $ST_EMISpath/indus_wood/stack_groups_indus_wood.ncf
setenv STK_GRPS_013 $ST_EMISpath/marine/stack_groups_marine.ncf
setenv STK_GRPS_014 $ST_EMISpath/nonroad_diesel/stack_groups_nonroad_diesel.ncf
setenv STK_GRPS_015 $ST_EMISpath/nonroad_gas/stack_groups_nonroad_gas.ncf
setenv STK_GRPS_016 $ST_EMISpath/other/stack_groups_other.ncf
setenv STK_GRPS_017 $ST_EMISpath/rail/stack_groups_rail.ncf
setenv STK_GRPS_018 $ST_EMISpath/rxfire/stack_groups_rxfire_20160101.ncf
setenv STK_GRPS_019 $ST_EMISpath/wildfire/stack_groups_wildfire_20160101.ncf

# Emission Rates for Offline Point Sources
setenv STK_EMIS_001 $EP_EMISpath/aircraft/inln_mole_aircraft_20160101.ncf
```

```
setenv STK_EMIS_002 $EP_EMISpath/dust/inln_mole_dust_20160101.ncf
setenv STK_EMIS_003 $EP_EMISpath/egu_biomass/inln_mole_egu_biomass_20160101.ncf
setenv STK_EMIS_004 $EP_EMISpath/egu_coal/inln_mole_egu_coal_20160101.ncf
setenv STK_EMIS_005 $EP_EMISpath/egu_natgas/inln_mole_egu_natgas_20160101.ncf
setenv STK_EMIS_006 $EP_EMISpath/egu_oil/inln_mole_egu_oil_20160101.ncf
setenv STK_EMIS_007 $EP_EMISpath/indus_biomass/inln_mole_indus_biomass_20160101.ncf
setenv STK_EMIS_008 $EP_EMISpath/indus_coal/inln_mole_indus_coal_20160101.ncf
setenv STK_EMIS_009 $EP_EMISpath/indus_gasoline/inln_mole_indus_gasoline_20160101.ncf
setenv STK_EMIS_010 $EP_EMISpath/indus_natgas/inln_mole_indus_natgas_20160101.ncf
setenv STK_EMIS_011 $EP_EMISpath/indus_oil/inln_mole_indus_oil_20160101.ncf
setenv STK_EMIS_012 $EP_EMISpath/indus_wood/inln_mole_indus_wood_20160101.ncf
setenv STK_EMIS_013 $EP_EMISpath/marine/inln_mole_marine_20160101.ncf
setenv STK_EMIS_014 $EP_EMISpath/nonroad_diesel/inln_mole_nonroad_diesel_20160101.ncf
setenv STK_EMIS_015 $EP_EMISpath/nonroad_gas/inln_mole_nonroad_gas_20160101.ncf
setenv STK_EMIS_016 $EP_EMISpath/other/inln_mole_other_20160101.ncf
setenv STK_EMIS_017 $EP_EMISpath/rail/inln_mole_rail_20160101.ncf
setenv STK_EMIS_018 $EP_EMISpath/rxfire/inln_mole_rxfire_20160101.ncf
setenv STK_EMIS_019 $EP_EMISpath/wildfire/inln_mole_wildfire_20160101.ncf

# Label Each Offline Point Emissions Stream
setenv STK_EMIS_LAB_001 EP_AIRCRAFT
setenv STK_EMIS_LAB_002 EP_DUST
setenv STK_EMIS_LAB_003 EP_EGU_BIOMASS
setenv STK_EMIS_LAB_004 EP_EGU_COAL
setenv STK_EMIS_LAB_005 EP_EGU_NATGAS
setenv STK_EMIS_LAB_006 EP_EGU_OIL
setenv STK_EMIS_LAB_007 EP_INDUS_BIOMASS
setenv STK_EMIS_LAB_008 EP_INDUS_COAL
setenv STK_EMIS_LAB_009 EP_INDUS_GASOLINE
setenv STK_EMIS_LAB_010 EP_INDUS_NATGAS
setenv STK_EMIS_LAB_011 EP_INDUS_OIL
setenv STK_EMIS_LAB_012 EP_INDUS_WOOD
setenv STK_EMIS_LAB_013 EP_MARINE
setenv STK_EMIS_LAB_014 EP_NONROAD_DIESEL
setenv STK_EMIS_LAB_015 EP_NONROAD_GAS
setenv STK_EMIS_LAB_016 EP_OTHER
setenv STK_EMIS_LAB_017 EP_RAIL
setenv STK_EMIS_LAB_018 EP_RXFIRE
setenv STK_EMIS_LAB_019 EP_WILDFIRE
```

Table S1. Examples of streams used for conventional and high-source-resolution cases.

Conventional Stream Label	Description	High Resolution Stream Label	Description
	Gridded		
GRIDDED_EMIS	All 2D gridded emissions merged except residential burning	GR_AGFIRE	Agricultural fires
GR_RES_FIRES	Residential wood burning	GR_AGRICULTURE	Agricultural processes (mainly ammonia)
		GR_AIRCRAFT	Airport support processes
		GR_COOKING	Cooking
		GR_DUST	Fugitive, road, agricultural and construction dust
		GR_EGU_BIOMASS	Biomass-fueled electric generation units
		GR_EGU_COAL	Coal-fueled electric generation units
		GR_EGU_NATGAS	Natural gas-fueled electric generation units
		GR_EGU_OIL	Oil-fueled electric generation units
		GR_INDUS_BIOMASS	Biomass-fueled industrial emissions
		GR_INDUS_COAL	Coal-fueled industrial emissions
		GR_INDUS_GASOLINE	Gasoline-fueled industrial emissions
		GR_INDUS_NATGAS	Natural gas-fueled industrial emissions
		GR_INDUS_OIL	Oil-fueled industrial emissions
		GR_INDUS_WOOD	Wood-fueled industrial emissions
		GR_MARINE	Ports and ships in domestic waters
		GR_NONROAD_DIESEL	Nonroad mobile diesel
		GR_NONROAD_GAS	Nonroad mobile gasoline
		GR_ONROAD_DIESEL	Onroad diesel
		GR_ONROAD_GAS	Onroad gas
		GR_OTHER	Other
		GR_RAIL	Rail
		GR_RES_DIESEL	Residential diesel burning
		GR_RES_OPENFIRE	Residential open fires
		GR_RES_WOOD	Residential wood stoves
		GR_RXFIRE	Prescribed Fires
		GR_WILDFIRE	Wildfires
	Point		
PT_EGU	Electric generating units	EP_AIRCRAFT	Aircraft landing and takeoff
PT_NONEGU	Non electric-generating industry	EP_DUST	Fugitive, agricultural and construction dust
PT_OILGAS	Oil and gas industry relevant	EP_EGU_BIOMASS	Biomass-fueled electric generating units
PT_CMV	Marine	EP_EGU_COAL	Coal-fueled electric generating units
PT_OTHER	Other	EP_EGU_NATGAS	Natural gas-fueled electric generating units
PT_OTHFIRES	Other fires	EP_EGU_OIL	Oil-fueled electric generating units
PT_FIRES	Wildfires	EP_INDUS_BIOMASS	Biomass-fueled industrial processes
PT_AGFIRES	Agricultural fires	EP_INDUS_COAL	Coal-fueled industrial processes
		EP_INDUS_GASOLINE	Gasoline-fueled industrial processes
		EP_INDUS_NATGAS	Natural gas-fueled industrial processes
		EP_INDUS_OIL	Oil-fueled industrial processes
		EP_INDUS_WOOD	Wood-fueled industrial processes
		EP_MARINE	Internaitonal shipping
		EP_NONROAD_DIESEL	Nonroad mobile diesel
		EP_NONROAD_GAS	Nonroad mobile gasoline
		EP_OTHER	Other
		EP_RAIL	Rail
		EP_RXFIRE	Prescribed fires
		EP_WILDFIRE	Wildfires

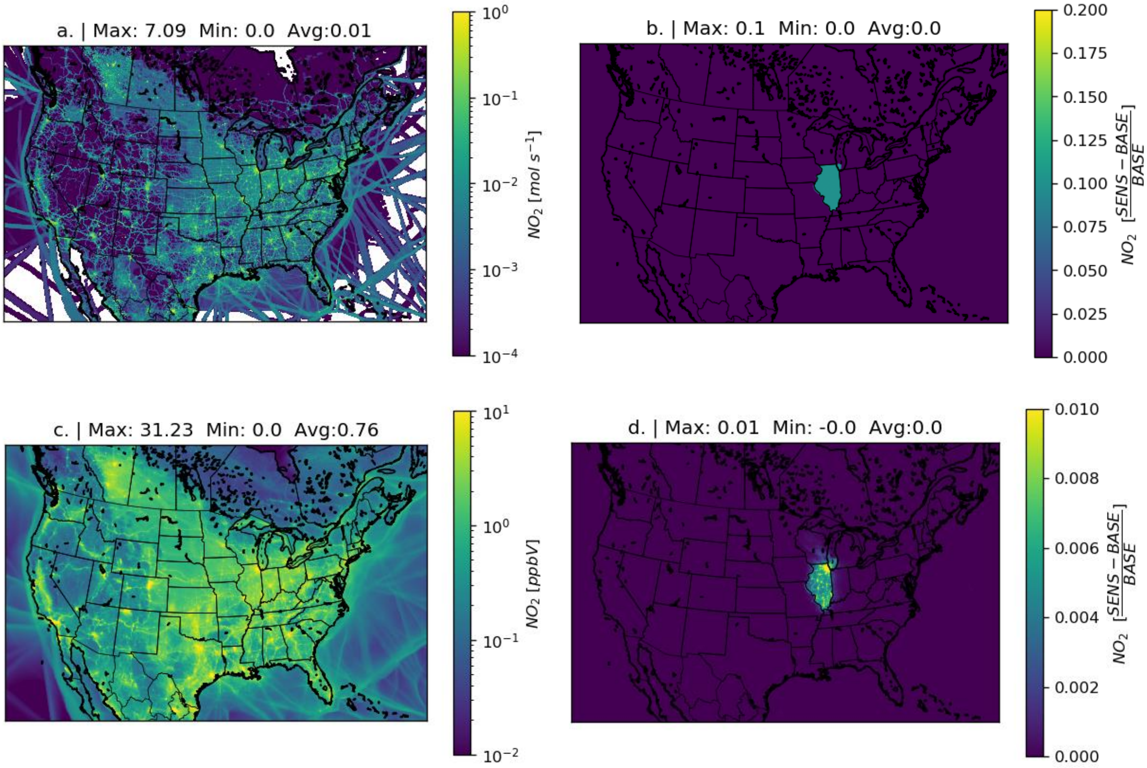

Figure S1. a) Average 1-hr NO_2 emissions input for June 3-10, 2016. b) Change in 1-hr NO_2 emissions for CMAQ run with ECI shown in section S2. c) Average NO_2 surface concentrations predicted by CMAQ for June 3-10, 2016. d) Change in average NO_2 concentration for ECI shown in section S2.

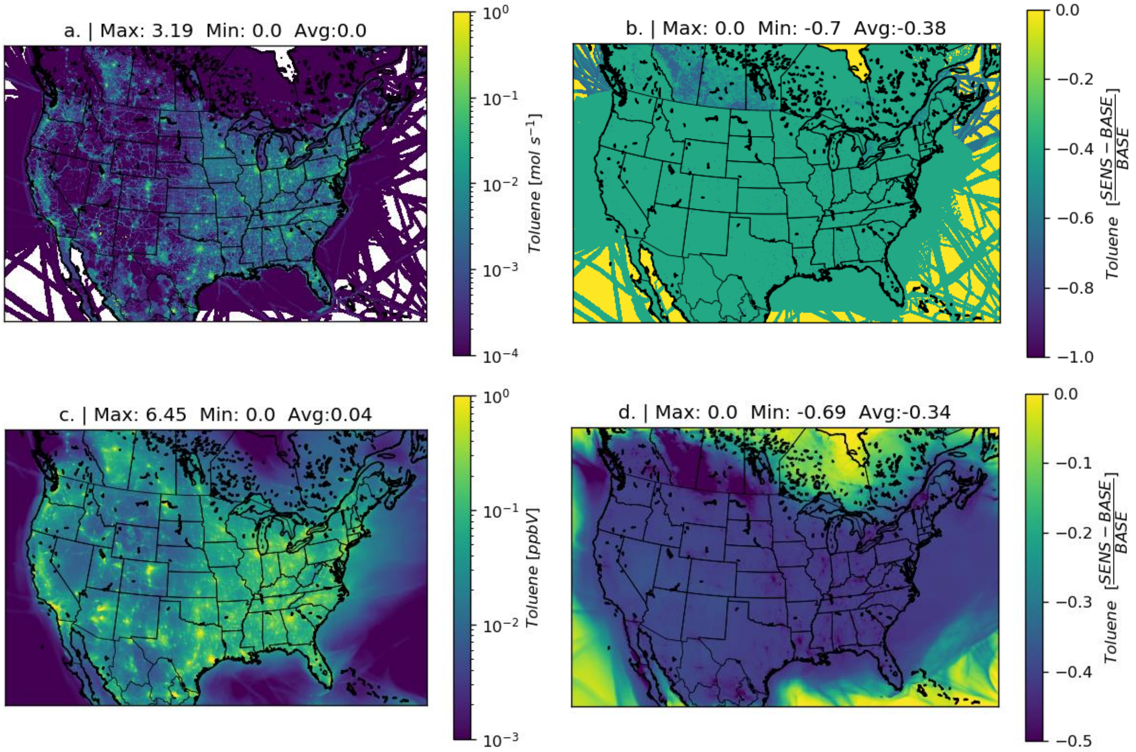

Figure S2. a) Average 1-hr TOL emissions input for June 3-10, 2016. b) Change in 1-hr TOL emissions for CMAQ run with ECI shown in section S3. c) Average TOL surface concentrations predicted by CMAQ for June 3-10, 2016. d) Change in average TOL concentration for ECI shown in section S3.

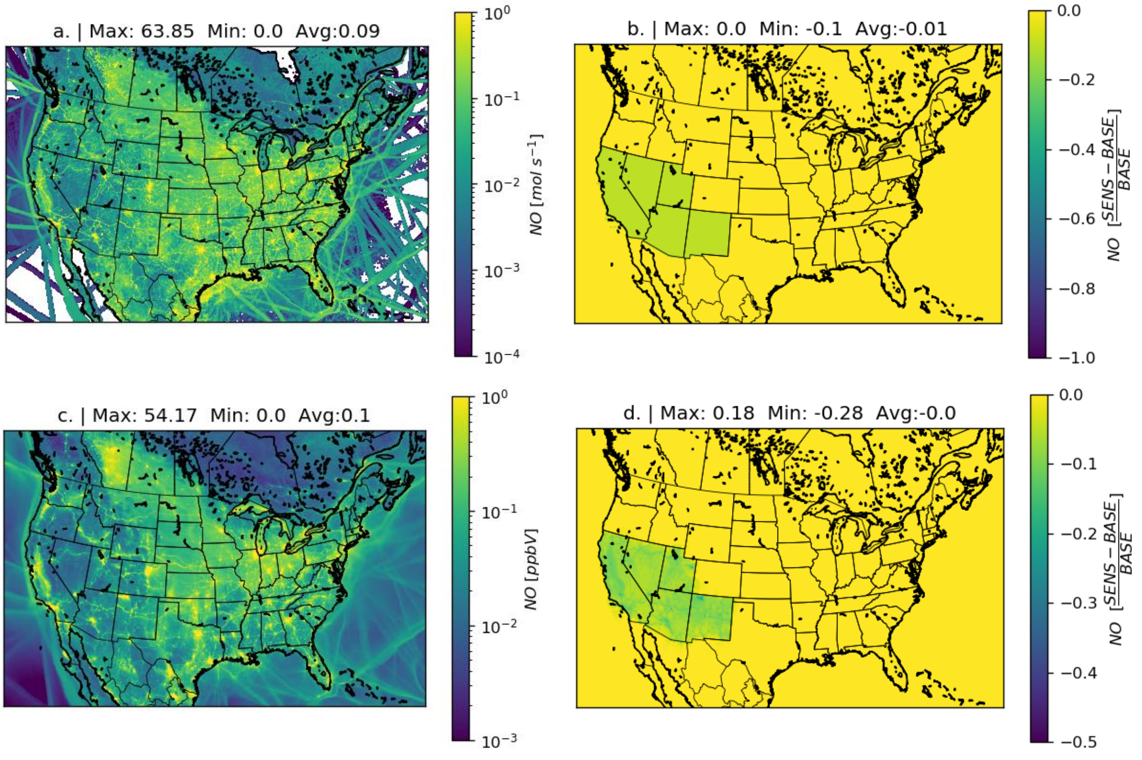

Figure S3. a) Average 1-hr NO emissions input for June 3-10, 2016. b) Change in 1-hr NO emissions for CMAQ run with ECI shown in section S3. c) Average NO surface concentrations predicted by CMAQ for June 3-10, 2016. d) Change in average NO concentration for ECI shown in section S3.
